# Neurogenesis and neuroplasticity activities of natural products: therapeutic potential in Alzheimer’s disease

**DOI:** 10.3389/fphar.2026.1863841

**Published:** 2026-08-05

**Authors:** Abeer M. Shaheen, Mai H. A. Mousa, Merhan O. Hindam, Ahmed M. El-Dessouki, Sally A. Fahim, Dina M. Hal, Sadek Ahmed, Riham A. El-Shiekh, Hazim O. Khalifa

**Affiliations:** 1 Department of Pharmacology and Toxicology, Faculty of Pharmacy, Heliopolis University, Cairo, Egypt; 2 Pharmaceutical Chemistry Department, Faculty of Pharmacy and Drug Technology, Egyptian Chinese University, Cairo, Egypt; 3 Department of Pharmacology and Toxicology, Faculty of Pharmacy, Cairo University, Cairo, Egypt; 4 Pharmacology and Toxicology Department, Faculty of Pharmacy, Ahram Canadian University, Giza, Egypt; 5 Department of Biochemistry, School of Pharmacy, Newgiza University (NGU), Giza, Egypt; 6 Department of Pharmacognosy, Faculty of Pharmacy, Suez Canal University, Ismailia, Egypt; 7 Department of Pharmaceutics and Industrial Pharmacy, Faculty of Pharmacy, Cairo University, Cairo, Egypt; 8 Department of Pharmacognosy, Faculty of Pharmacy, Cairo University, Cairo, Egypt; 9 Department of Veterinary Medicine, College of Agriculture and Veterinary Medicine, United Arab Emirates University, Al Ain, United Arab Emirates; 10 United Arab Emirates University (UAEU) Center for Public Policy and Leadership, United Arab Emirates University, Al Ain, United Arab Emirates

**Keywords:** Alzheimer’s disease, BDNF signaling, natural products, neurogenesis, neuroplasticity

## Abstract

Alzheimer’s disease (AD) is a progressive neurodegenerative disorder characterized by memory loss, cognitive decline, and behavioral disturbance, largely associated with amyloid-β accumulation, tau protein pathology, synaptic dysfunction, oxidative stress and neuroinflammation. Previous research emphasizes the critical roles of impaired neurogenesis and neuroplasticity in cognitive dysfunction and neurodegenerative disorders, particularly AD. This review highlights the therapeutic potential of natural products in restoring these processes. It offers a comprehensive overview of the molecular mechanisms underlying neurogenesis and synaptic plasticity, with a focus on key regulatory pathways, such as BDNF/TrkB, ERK/CREB, PI3K/Akt and neurotransmitter signaling cascades. It also shows how the disruption of these regulatory pathways, caused by AD-associated pathological factors, results in reduced neuronal regeneration and synaptic connectivity. Furthermore, it summarizes preclinical data showing that various phytochemical compounds, such as ginsenosides, epigallocatechin gallate, curcumin, resveratrol, and flavonoids can improve neural stem cell proliferation, promote neuronal differentiation, and enhance synaptic function. Despite the promising pre-clinical studies results, clinical studies evaluating natural products remain limited due to poor bioavailability, inconsistent efficacy, and methodological constraints in human studies. To overcome these limitations, new strategies such as nanotechnology-based delivery systems and advanced brain targeting approaches are being developed. Overall, natural products represent promising candidates for managing neurogenesis and neuroplasticity in AD, although further clinical studies are required to validate their therapeutic efficacy.

## Graphical Abstract

**Figure:**
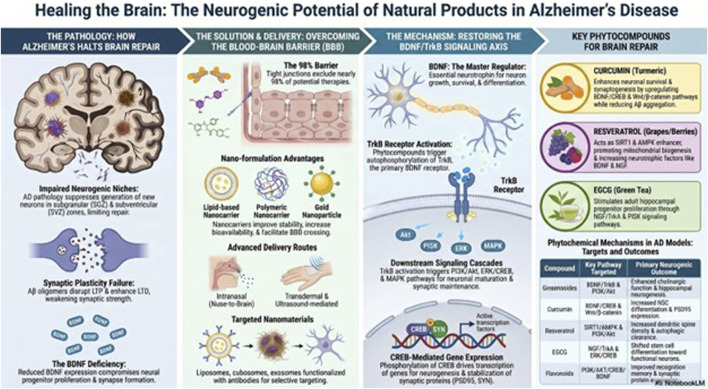
ToC highlight: The observer effect in biological imaging: small-molecule fluorescent probes perturb the systems they report on through Ca^2+^ buffering, organelle interference, dark toxicity, and phototoxicity. Label-free autofluorescence provides a minimal-perturbation benchmark; future probes must be designed as bioactive entities, not merely optical tools.

## 1 Introduction

Alzheimer’s disease (AD) is a progressive neurodegenerative disorder and the leading cause of dementia worldwide. It is characterized by memory loss, cognitive decline, and behavioral disturbances (Mhaske et al., 2025). Pathologically, AD is associated with the accumulation of amyloid-β (Aβ) plaques and neurofibrillary tangles composed of hyperphosphorylated tau protein (Abdulkhaliq et al., 2026). Synaptic loss and widespread neuronal degeneration are among the major hallmarks of AD (Boon et al., 2025). Although several medications are currently approved for the treatment of AD, their effects are primarily symptomatic and do not halt or reverse neuronal loss (Kumari et al., 2025). Therefore, identifying novel therapeutic strategies that promote brain regeneration and target the underlying mechanisms of neurodegeneration is critically important (Faraji et al., 2025). In recent years, increasing attention has been directed toward the concepts of neurogenesis and neuroplasticity in the adult brain (Rahman and Prabhakar, 2025). These biological processes are essential for learning, memory, and cognitive flexibility and are required for maintaining normal brain function. Impairments in neurogenesis and neuroplasticity have been closely linked to cognitive dysfunction and neurodegenerative disorders, particularly AD (Zhang et al., 2025a).

Neurogenesis refers to the generation of new neurons from neural stem or progenitor cells (Jang et al., 2025). Contrary to earlier assumptions, neurogenesis persists throughout adulthood but is restricted to specific brain regions known as neurogenic niches (Vaz et al., 2022). These niches provide specialized microenvironments that maintain neural stem cells (NSCs) and tightly regulate their capacity to generate new neurons across the lifespan (Gonzalez-Perez et al., 2012). The primary neurogenic niches in the adult brain are the subgranular zone (SGZ) of the hippocampal dentate gyrus and the subventricular zone (SVZ) lining the lateral ventricles. In the SGZ, radial glia-like stem cells divide to produce intermediate progenitor cells and neuroblasts, which ultimately mature into granule neurons that integrate into hippocampal circuits involved in learning and memory. In the SVZ, astrocyte-like stem cells generate transit-amplifying progenitors and migratory neuroblasts (Conover and Todd, 2017).

Adult neurogenesis is a highly organized and multistep process. It begins with the proliferation of neural stem and progenitor cells, followed by their differentiation into immature neurons (Gallardo-Caballero et al., 2026). These immature neurons then migrate to their appropriate locations, undergo maturation, and become functionally integrated into pre-existing neural circuits. Successful integration enables these neurons to participate in synaptic transmission and cognitive processes (Simard et al., 2025). Each stage of neurogenesis is regulated by numerous factors, including growth factors, neurotransmitters, hormones, and environmental stimuli (Choquet de Isla et al., 2025). In Alzheimer’s disease, adult neurogenesis is severely compromised by multiple interconnected pathological pathways. Aβ toxicity, tau pathology, oxidative stress, and chronic neuroinflammation have been shown to impair neural progenitor cell proliferation, inhibit neuronal differentiation, and disrupt the survival and integration of newly generated neurons (Rodríguez and Verkhratsky, 2011). Consequently, hippocampal regenerative capacity is compromised, further contributing to memory deficits and cognitive decline observed in patients with AD (Ma et al., 2025).

Neuroplasticity refers to the brain’s cognitive ability to adapt its shape and function in response to experience, learning, and external environmental factors. It is a permanent property of the nervous system and is crucial for memory development, learning, and adaptation (Kalra et al., 2025). Neuroplasticity encompasses both structural and functional changes at the synaptic and neuronal levels. Synaptic plasticity, one of its most important forms, is primarily represented by long-term potentiation (LTP) and long-term depression (LTD.) (Di Filippo et al., 2009). LTP refers to a sustained increase in synaptic strength, whereas LTD. represents a long-lasting decrease in synaptic efficacy. These mechanisms are widely considered the cellular and molecular foundations of learning and memory. In AD, synaptic plasticity is markedly impaired; Aβ oligomers disrupt LTP and enhance LTD., leading to synaptic dysfunction and cognitive deficits (Zhang et al., 2022a).

Natural products have demonstrated the potential to enhance neural stem cell proliferation, promote neuronal differentiation, and improve the survival and integration of newly generated neurons. Therefore, natural products represent promising candidates for the development of novel therapeutic strategies aimed at restoring neurogenesis and neuroplasticity in AD. The present review aims to comprehensively examine the effects of natural products on neurogenesis and neuroplasticity and to evaluate their therapeutic potential in AD. To provide a rigorous translational assessment, this review explicitly categorizes findings into three distinct evidentiary tiers: (1) *mechanistic plausibility*, evaluated via *in silico* target-binding frameworks; (2) *preclinical efficacy*, demonstrated through cellular and animal disease models; and (3) *clinically meaningful therapeutic outcomes*, validated through objective cognitive endpoints in human clinical trials. Maintaining this distinction is vital to bridge the translational gap without over-interpreting experimental biomarkers as proven clinical treatments.

## 2 Search strategy

A narrative literature search was conducted to identify relevant studies on the neurogenesis- and neuroplasticity-enhancing effects of natural products in Alzheimer’s disease. The search was performed in the following electronic databases: PubMed/MEDLINE, Scopus, Web of Science, and Google Scholar for articles published up to April 2026. The search strategy was done using the following terms: “Alzheimer’s disease” OR “ neurogenesis” OR “neuroplasticity” OR “ natural products” “phytochemicals” OR “synaptic plasticity” OR “brain-derived neurotrophic factor” OR “related signaling pathways such as BDNF, TrkB, CREB, PI3K/Akt, and ERK”.

The retrieved literature was filtered using a stringent inclusion and exclusion protocol to ensure the critical integration of high-quality data. Studies were strictly included if they met the following criteria: (1) Investigations on natural products, phytochemicals, or plant-derived compounds in relation to neurogenesis, neuroplasticity, synaptic function, or cognitive outcomes in Alzheimer’s disease or relevant experimental models, (2) Original preclinical studies, clinical studies, or relevant review articles, and (3) Published in peer-reviewed journals. Conversely, literature was excluded if it consisted of abstracts without full-text access, conference proceedings, or informal editorials, or if the study focused exclusively on unrelated neurological disorders lacking direct mechanistic relevance to Alzheimer’s disease. Reference management software was used to remove duplicate records, resolve formatting inconsistencies, and ensure rigorous indexing.

## 3 Dysregulation of neurogenesis and plasticity in AD

AD is marked by severe impairments in adult hippocampal neurogenesis and synaptic plasticity, which are major contributors to progressive cognitive decline. AD causes progressive cognitive decline and neuronal loss, partly due to the buildup of Aβ plaques and tau tangles (Gao et al., 2022; Dwamena et al., 2025).

### 3.1 Restoring BDNF signaling to rescue neurogenesis and synaptic plasticity in AD

Among the molecular regulators implicated, brain-derived neurotrophic factor (BDNF) has emerged as a central therapeutic target, as its restoration consistently ameliorates neurogenic and synaptic deficits. BDNF expression is substantially reduced in the hippocampus and entorhinal cortex of AD brains, largely due to Aβ oligomer toxicity and tau pathology that disrupt TrkB–ERK–CREB signaling and suppress activity-dependent BDNF transcription (Gao et al., 2022). This deficiency compromises neural progenitor proliferation, neuronal maturation, and synapse formation, as evidenced by marked reductions in doublecortin-positive cells, NeuroD1 expression, and synaptic markers such as PSD95 (Tang et al., 2025). BDNF signaling through TrkB activates PI3K/Akt, ERK/CREB, and mTOR pathways, thereby promoting neuronal differentiation, synaptic maintenance, and inhibition of tau hyperphosphorylation, while also modulating amyloid processing toward non-amyloidogenic pathways (Thomson et al., 2024).

In multiple transgenic AD mouse models, including APP/PS1 and regulatable Transgenic 4,510 (rTg4510), hippocampal BDNF overexpression restores neurogenesis- and plasticity-related gene programs, improves spatial memory, and attenuates neurodegenerative features independently of amyloid or tau burden (Alqahtani et al., 2025). In AD patients, reduced BDNF levels correlate with cognitive impairment and are further influenced by the valine-66-methionine (Val66Met) polymorphism, which impairs BDNF secretion (Yang et al., 2015). Although challenges such as blood–brain barrier delivery remain, emerging strategies, including gene therapy, TrkB agonists, and lifestyle interventions that elevate endogenous BDNF, highlight the translational potential of targeting BDNF to counteract neurogenesis and plasticity deficits in AD beyond conventional amyloid- and tau-focused approaches (Koppensteiner et al., 2016).

### 3.2 Enhancing neurotransmitter release and synaptogenesis to counteract neurogenesis and plasticity deficits in AD

AD is characterized by impaired neurotransmitter release and synapse loss emerging as key drivers of cognitive decline. In the brains of APP/PS1 transgenic mice, Aβ oligomers directly bind syntaxin 1a and disrupt soluble N-ethylmaleimide-sensitive factor attachment protein receptor (SNARE)-mediated vesicle release, leading to impaired neurotransmitters release (Zhang et al., 2021). Both Aβ oligomers and hyperphosphorylated tau compromise presynaptic vesicle dynamics and postsynaptic integrity. This disruption leads to reduced glutamatergic and GABAergic transmission, diminished synaptic density, and LTP. Concurrently, neurogenesis in the hippocampus is markedly suppressed, further limiting circuit adaptability and memory encoding. Aβ–treated mice exhibited a transient reduction in synaptic plasticity over several hours, accompanied by an increase in neurotransmitter release (Hörtnagl et al., 2013). Experimental studies in transgenic AD mouse models reveal that targeted enhancement of neurotransmitter release and synapse formation—through neurotrophic support, glutamatergic modulation, or antidepressant-based interventions—can restore synaptic signaling and improve cognitive performance independently of amyloid or tau burden (Abu-Taweel et al., 2013; Li et al., 2025). Pharmacological agents such as curcumin, selective serotonin reuptake inhibitors, and NMDA receptor modulators promote neurogenesis, synaptic protein expression, and functional connectivity by increasing the neurotransmitters (Lutzu and Castillo, 2021; O’Day, 2023; Jiménez-Balado and Eich, 2021).

Beyond amyloid and tau pathology, dysregulation of neurotransmitter release and synapse formation in AD is driven by several interconnected mechanisms. Aβ and tau-mediated disruption of calcium homeostasis alters presynaptic release probability through abnormal activation of voltage-gated calcium channels, N-methyl-D-aspartate (NMDA) receptors, and endoplasmic reticulum calcium leakage, leading to synaptic fatigue and excitotoxic stress (Grinberg, 2023). An imbalance between excitatory and inhibitory signaling further destabilizes neural networks in AD. Loss of GABAergic interneurons leads to hyperexcitability and abnormal increases in glutamate release (Wang et al., 2020). Glial cells also contribute to synaptic dysfunction: activated microglia enhance complement-mediated synaptic pruning, while impaired astrocytes fail to efficiently clear excess glutamate, promoting synapse loss (Pumo et al., 2022). In addition, mitochondrial dysfunction at synapses reduces ATP production, limiting the energy required for neurotransmitter release and structural maintenance (Klempin et al., 2010). Epigenetic and activity-dependent gene programs that normally support synapse formation are also weakened (Verdurand and Zimmer, 2017). Together, these alterations progressively impair synaptic connectivity and plasticity.

Collectively, these findings highlight enhancement of neurotransmitter release and synaptogenesis as promising, pathology-independent strategies to counteract early synaptic failure in AD, with encouraging translational signals emerging from mild cognitive impairment and early-stage patient cohorts.

### 3.3 Restoring hippocampal neurogenesis in AD through enhanced 5-HT1A receptor signaling

Serotonin regulates adult hippocampal neurogenesis through receptor subtype–specific mechanisms acting at distinct developmental stages. Activation of 5-hydroxytryptamine receptor (5-HT1A) receptors promotes precursor self-renewal, whereas 5-HT2/5-HT2C signaling influences proliferation and neuronal differentiation; sustained serotonergic stimulation enhances survival and maturation of postmitotic neurons, leading to a net increase in neurogenesis despite minimal acute effects on proliferation (Segi-Nishida, 2017). In Alzheimer disease, marked impairment of hippocampal neurogenesis and synaptic plasticity is partly linked to reduced 5-HT1A receptor signaling. Consistently, postmortem and neuroimaging studies show a significant decline in hippocampal 5-HT1A receptor density in individuals with mild cognitive impairment and Alzheimer disease, correlating with pyramidal neuron loss and cognitive decline (Mori et al., 2014).

Preclinical studies indicate that pharmacological or genetic enhancement of 5-HT1A receptor activity robustly stimulates hippocampal neurogenesis. Chronic selective serotonin reuptake inhibitor treatment increases proliferating and differentiating neural progenitors through autoreceptor desensitization and postsynaptic 5-HT1A activation, leading to upregulation of ERK/CREB signaling and BDNF expression (Sullivan et al., 2005). Selective and biased 5-HT1A agonists similarly enhance neurogenesis markers, synaptic proteins, and cognitive performance in aged and transgenic AD models, effects that appear independent of amyloid burden (Wu et al., 2024).

**FIGURE 1:**
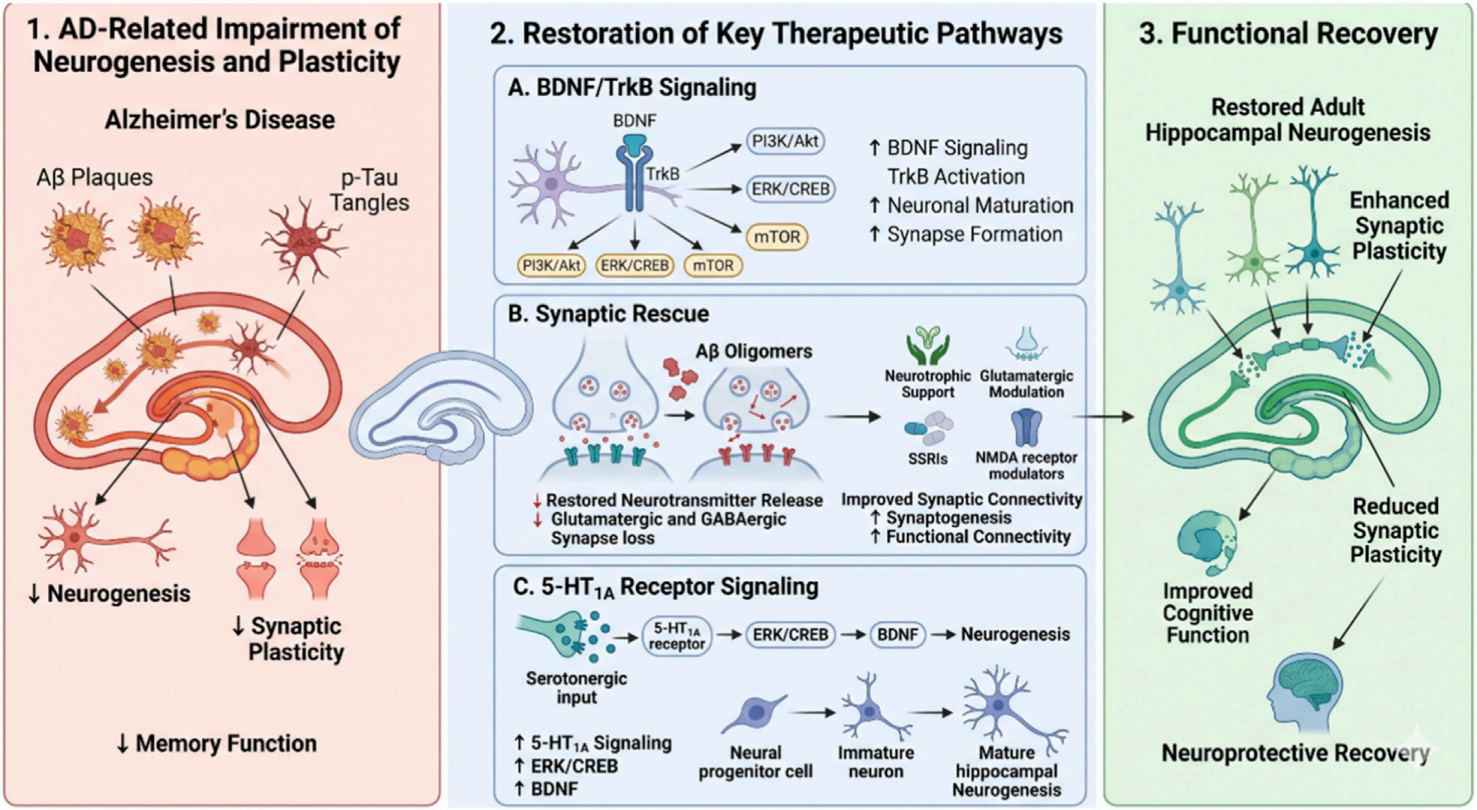
Mechanisms of neurogenesis and synaptic plasticity dysregulation in AD. AD involves impaired neurogenesis and synaptic plasticity, largely driven by Aβ accumulation, tau pathology, oxidative stress, and neuroinflammation. Reduced BDNF signaling disrupts TrkB–ERK–CREB pathways, limiting neural progenitor proliferation, neuronal differentiation, and synapse formation. AD pathology also impairs neurotransmitter release and synaptogenesis, partly through Aβ–mediated disruption of SNARE-dependent vesicle release, calcium dysregulation, glial-mediated synaptic pruning, and mitochondrial dysfunction. Additionally, reduced serotonergic signaling via 5-HT1A receptors contributes to impaired hippocampal neurogenesis and synaptic plasticity. Therapeutic strategies aimed at restoring BDNF signaling, enhancing neurotransmitter release, and activating 5-HT1A receptor pathways may help restore neuronal regeneration, synaptic connectivity, and cognitive function in AD.

In the brain of male rats, 5-HT1A receptors couple to Gi/o proteins, modulating cyclic AMP signaling and activating ERK pathways that promote neuronal maturation, migration, and survival (Aguiar et al., 2023). Astrocytic 5-HT1A receptors further contribute to excitation–inhibition balance and plasticity regulation (Polter and Li, 2010). Experimental evidence across multiple *in vivo* Alzheimer disease models demonstrates that increasing 5-HT1A receptor activity preserves progenitor pools and improves spatial memory by increasing BDNF and PSD95 protein levels (Verdurand and Zimmer, 2017). Although serotonergic neuron loss and receptor regulation present therapeutic challenges, targeted modulation of 5-HT1A signaling remains a promising strategy to counteract hippocampal neurogenesis deficits in AD. While these core neurotrophic, neurotransmitter, and serotonergic pathways establish the primary molecular framework for structural plasticity, they also serve as the precise upstream therapeutic targets for specific plant-derived compounds. Consequently, the subsequent section transitions from these isolated pathways to evaluate how individual phytochemicals distinctively modulate these exact signaling nodes to drive functional neurogenic repair (Figure 1).

## 4 Phytochemical compounds with neurogenic and neuroplasticity-enhancing activities: evidence from pre-clinical study

**FIGURE 2:**
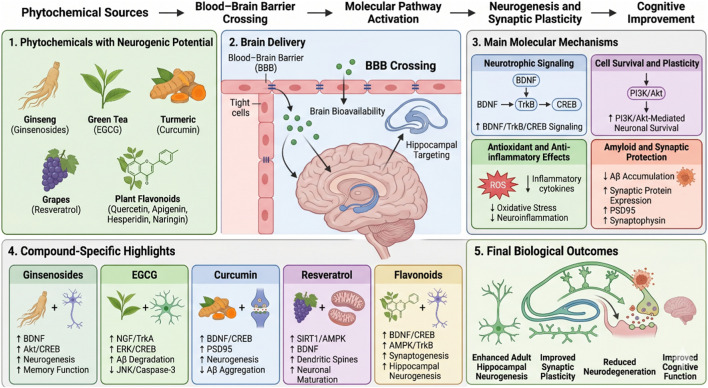
Phytochemicals promote neurogenesis and synaptic plasticity in AD. Several plant-derived phytochemicals enhance neurogenesis and synaptic plasticity in preclinical AD models. Ginsenosides stimulate hippocampal neurogenesis via BDNF/TrkB–PI3K/Akt–CREB signaling. EGCG promotes neural progenitor proliferation and neuroplasticity through NGF/TrkA, ERK/CREB, and PI3K/Akt pathways, while reducing amyloid burden and inflammation. Curcumin improves neuronal survival and synaptic plasticity via BDNF/CREB, PI3K/Akt, and Wnt/β-catenin signaling. Resveratrol enhances neurogenesis through SIRT1, AMPK, and PI3K/Akt pathways, increasing neurotrophic factors. Flavonoids such as quercetin, apigenin, and hesperidin further promote neural stem cell proliferation and synapse formation through CREB/BDNF-dependent mechanisms, collectively supporting neuronal regeneration and cognitive function in AD models.

Phytochemicals have attracted attention for their ability to cross the blood-brain barrier (BBB) and their pleiotropic effects in neuronal disorders (Adhikary et al., 2025). A growing body of preclinical studies indicates that certain plant-derived phytocompounds can effectively cross the BBB and modulate synaptic plasticity, dendritic density, and neurogenesis through multiple molecular mechanisms, including antioxidant, anti-inflammatory, and neurotrophic effects (Shoaib et al., 2023). Based on pre-clinical investigations, key phytocompounds with neurogenic and synaptic plasticity-enhancing effects against AD, such as ginsenosides found in ginseng, epigallocatechin gallate (EGCG) found in green tea, curcumin found in turmeric, resveratrol found in grapes, and flavonoids found in many plants, such as quercetin, apigenin, and hesperidin (Szala-Rycaj et al., 2025). This review highlights phytocompounds and their mechanisms of neurogenicity and plasticity-enhancing activities in various pre-clinical models, as shown in Table 1 and Figure 2.

**TABLE 1:** Examples of phytochemicals with neurogenic and plasticity-enhancing activity in AD.

Compounds	Model	Outcomes	References
Ginsenosides	Middle cerebral artery occlusion (MCAO) followed by reperfusion in rats	Upregulate BDNF/TrkB pathway Enhance neuronal survival and plasticity Activate PI3K/Akt pathway	Liu et al. (2015)
	Chronic unpredictable mild stress	Increase pAkt, pERK1/2, and pCREB expression Activate BDNF-TrkB signaling	Jiang et al. (2021)
	MCAO/R model, cell culture	Activate BDNF/Akt/CREB signaling Enhance neurogenesis and oligodendrogenesis Reduce neuronal loss	Zhu et al. (2021)
	APP/PS1 mice	Enhance neurogenesis and synaptogenesis Upregulate CREB/BDNF pathway	She et al. (2024)
EGCG	APP/PS1 transgenic mice	Increase NGF/proNGF ratio Activate TrkA/ERK/CREB pathway Reduce Aβ plaques, JNK/cleaved caspase-3 expression	Liu et al. (2014)
		Promote the proliferation of adult hippocampal progenitor cells Differentiate stem cells into functional neurons rather than glial cells	Wang et al. (2012)
	Ovariectomized mice fed a high-fat diet	Regulation of the gut-brain axis Activate ERK/PI3K pathway Improve neurogenesis and neuroplasticity Reduce Aβ degradation	Qu et al. (2023)
	Cell culture	Activate MAPK/Erk and PI3K Signaling pathways in astrocytes Increase astrocyte secretion of NEP facilitate Aβ degradation in cortical astrocytes	Yamamoto et al. (2017)
	High‐fat–and high‐fructose‐induced cognitive defects in c57BL/6J mice	Activate the Erk/CREB/BDNF pathway Promote neurogenesis and synaptogenesis Downregulate MAPK/NF-kB cascade Reduce neuroinflammation	Mi et al. (2017)
Curcumin	hypoxic-brain injury model in C57BL/6J mice	preserve synaptic plasticity and neurogenesis, by increasing BDNF and PSD95 expression	Li et al. (2025)
	*In vitro* and *in vivo* APP/PS1 mouse model	Promote NSCs differentiation into functional neurons Reduced Aβ aggregation in the hippocampus and cortex Enhance neurogenesis by upregulating the BDNF/CREB pathway	Chen et al. (2025)
	Aβ_1–42_ mice model of AD	Increasing hippocampal BrdU-positive cells regulate PI3K/Akt pathway Inhibit GSK3β Activate wnt/β-catenin and CREB/BDNF pathway	Lou et al. (2024)
	Cisplatin-induced chemo-brain model in rats	Activate autophagy via elevation of AMPK-JNK signaling Downregulation of the mTOR and Bcl-2 expression Inhibiting apoptosis	Yi et al. (2020)
Resveratrol	Balb/C mice model of AD	Increase BrdU-positive cells, Akt, and PKC proteins Promote neurogenesis and increase the number of dendritic spines Augment neuronal precursor cells differentiating into mature neurons	Torres-Pérez et al. (2015)
	Mice models of AD	Promote neurogenesis via upregulation of SIRT1/wnt signaling Upregulation of the AMPK/BDNF pathway Improving autophagic clearance of protein aggregates	Surya et al. (2023)
	hUC-MSCs culture cell	Upregulate the PI3K/Akt/BDNF pathway Reduce neuroinflammation Boost the expression of BDNF, NT-3, and NGF.	Wang et al. (2018)
Quercetin	Aβ-induced model of AD	Enhance NSCs’ proliferation into mature neurons Increase the number of BrdU/NeuN-positive cells Upregulate BDNF, NGF, CREB, and EGR-1genes	Karimipour et al. (2019)
	Double blow model-induced cognitive impairment and schizophrenia-like mice	Activate the PI3K/AKT/CREB/BDNF signaling pathway increase the expression of synaptic plasticity proteins SYN and PSD95	Yang et al. (2025)
Apigenin	Pentylenetetrazol-treated mice	Promoting hippocampal neurogenesis by increasing phosphorylation of BDNF/CREB proteins	Sharma et al. (2020)
	Aβ25-35-induced cellular stress and cognitive impairment in SH-SY5Y cells	Increase neurite outgrowth in cultured neurons Augment TrkB/BDNF expression	Gao et al. (2023)
Hesperidin	Cell culture of NSCs	Restore hippocampal neurogenesis Activate AMPK/BDNF/TrkB/CREB signaling	Lee et al. (2022)
	*In vitro* and *in vivo* model	Enhance the synaptogenic capacity via increased secretion of TGF-β1	Matias et al. (2017)
Naringin	Utero-placental insufficiency model	Enhance cholinergic activity Upregulate CREB/BDNF signaling	Nemati et al. (2025)
	Trimethyltin-induced cognitive dysfunction in rats b	Inhibit expression of p-tau, Aβ, presenilin 1, and acetylcholinesterase Reduce oxidative and nitrosative stress	Faryadras et al. (2025)

### 4.1 Ginsenosides

Ginsenosides are the main bioactive compounds derived from the ginseng plant and exhibit neuroprotective action in the treatment of AD (Mehrnoosh et al., 2025). A previous study showed that ginsenoside administration protects the brain from Aβ-induced neurotoxicity, enhances cholinergic function, and stimulates hippocampal neurogenesis, thereby improving cognitive and memory function (Kim et al., 2018). Furthermore, it has been demonstrated that ginsenosides enhance neurogenesis by upregulating BDNF, which in turn binds to tropomyosin receptor kinase B (TrkB) receptor, resulting in autophosphorylation of receptors and activating several signaling pathways required for neuronal survival and plasticity, such as phospholipase Cγ2 (PLCγ2) that modulates calcium-dependent plasticity, Phosphoinositide 3-kinase (PI3K)/Protein kinase B (Akt) pathway that promotes neuronal (Liu et al., 2015; Jiang et al., 2021). Additionally, in the middle cerebral artery occlusion/reperfusion stroke model in rats and in PC12 cells, ginsenosides exacerbate neurogenesis and oligodendrogenesis by increasing BDNF levels and activating the Akt/cyclic AMP response element-binding protein (CREB) signaling cascade (Zhu et al., 2021). Similarly, ginsenoside RK3 enhances neurogenesis in AD through activation of the CREB/BDNF pathway in Aβ-inuced AD model (She et al., 2024).

### 4.2 Epigallocatechin gallate (EGCG)

EGCG, a natural catechin derived from green tea, has high BBB permeability and powerful antioxidant properties and has been shown to be effective in alleviating AD symptoms (Cascella et al., 2017). Prior pre-clinical study reported that EGCG reduces the risk of cognitive deficits by enhancing neurogenesis through activation of the nerve growth factor (NGF) by increasing NGF/proNGF ratio, increasing phosphorylation of TrkA signaling pathway following phosphorylation of extracellular signal-regulated kinase (ERK)/CREB pathway, decreasing Aβ plaques, and suppressing c-Jun-N-terminal kinase (JNK)/cleaved caspase-3 expression in amyloid precursor protein (APP)/presenilin-1(PS1) transgenic mice (Liu et al., 2014). Moreover, EGCG stimulates neurogenesis by promoting the proliferation of adult hippocampal progenitor cells, directing stem cells toward functional neurons rather than glial cells (Wang et al., 2012). Furthermore, EGCG exerts its neurogenic and plasticity-enhancing effects by activating mitogen-activated protein kinase (MAPK)/Erk and PI3K Signaling pathways in astrocytes, thereby increasing astrocyte secretion of neprilysin (NEP) and facilitating Aβ degradation in cortical astrocyte cell culture (Yamamoto et al., 2017). EGCG also restored spatial and episodic memory deficits and morphological abnormalities observed in ovariectomized mice fed a high-fat diet, via regulation of the gut-brain axis and improvements in neurogenesis and neuroplasticity (Qu et al., 2023). Additionally, EGCG promotes neurogenesis via upregulation of Erk/CREB/BDNF and insulin receptor substrate-1 (IRS-1)/AKT signaling pathways, and suppressing hippocampal neuroinflammation by downregulating MAPK/nuclear factor kappa B (NF-κB) cascade, resulting in a significant reduction in inflammatory cytokine levels such as tumor necrosis factor-alpha (TNF-α), along with neutralizing oxidative stress and mitochondrial dysfunction in high-fat and high-fructose diet (HFFD)-induced cognitive impairment in c57BL/6J mice (Mi et al., 2017).

### 4.3 Curcumin

Curcumin is a polyphenolic phytochemical derived from Curcuma with powerful anti-inflammatory, antioxidant, and anti-apoptotic properties, as well as neurotrophic action (Armağan and Nazıroğlu, 2021). Curcumin mitigates cognitive impairment by preserving synaptic plasticity and neurogenesis, increasing BDNF and postsynaptic density protein 95 (PSD95) expression in the Thy1-YFP transgenic mouse model of AD (Li et al., 2025). Furthermore, curcumin increased NSCs differentiation into functional neurons *in vitro*, reduced Aβ aggregation in the hippocampus and cortex, and promoted neurogenesis by upregulating the BDNF/CREB pathway in the APP/PS1 transgenic mouse model (Chen et al., 2025). In addition, pre-clinical study revealed that curcumin improved hippocampal neuronal survival by increasing hippocampal BrdU-positive cells, regulating PI3K/Akt, inhibiting glycogen synthase kinase-3β (GSK3β), which is the negative regulator of wingless-related kinase (wnt)/beta-catenin (β-catenin), and boosting CREB/BDNF pathway that is crucial for self-repair mechanisms (Lou et al., 2024). Additionally, curcumin promoted neurogenesis and synaptogenesis by activating autophagy and inhibiting apoptosis in the cisplatin-induced chemo-brain model, as evidenced by the remarkable elevation in AMP-activated protein kinase (AMPK)-JNK signaling and the notable downregulation of mammalian target of rapamycin (mTOR) and B-cell lymphoma 2 (Bcl-2) expression (Yi et al., 2020).

### 4.4 Resveratrol

Resveratrol is a phenolic bioactive compound known for its potent anti-inflammatory and antioxidant properties, which protect the brain against many neurodegenerative disorders (Islam et al., 2022). Resveratrol-treated BALB/C mice showed significant improvement in hippocampal neurogenesis by increasing BrdU-positive cells, upregulating Akt and protein kinase C (PKC) proteins that modulate neurogenesis, and increasing the number of dendritic spines, accompanied by an increase in neuronal precursor cells differentiating into mature neurons (Torres-Pérez et al., 2015). Previous study demonstrated that resveratrol is a sirtuin-1 (SIRT1) enhancer, which is the positive regulator of wnt signaling that is very important for neurogenesis, in addition to upregulation of the AMPK pathway, which, in turn, promotes mitochondrial biogenesis, enhances autophagic clearance of protein aggregates, and upregulates BDNF expression (Surya et al., 2023). Additionally, resveratrol exerts its neuroprotective effects against AD by upregulating the PI3K/Akt/BDNF pathway, reducing neuroinflammation, and protecting against synaptic loss 5. Moreover, resveratrol boosted the expression of BDNF, neurotrophin 3 (NT-3), and NGF, all of which are linked to neurogenesis, survival, learning, and memory (Wang et al., 2018).

### 4.5 Flavonoids (quercetin, apigenin, hesperidin)

Multiple classes of flavonoids have been reported to promote neurogenesis and synaptogenesis by stimulating dendritic growth and synaptic formation, and to activate neurotrophic signaling, thereby exhibiting neuroprotective potential against AD (Cichon et al., 2020). Prior investigation demonstrated that quercetin improved neurogenesis in the dentate gyrus (DG) of Aβ-treated rats by enhancing NSCs proliferation into mature neurons, increasing the number of BrdU/NeuN-positive cells, and upregulating BDNF, NGF, CREB, and early growth response protein 1 (EGR-1) genes, which are involved in regulating neurogenesis (Karimipour et al., 2019). Furthermore, quercetin improved recognition and spatial memory in schizophrenic mice by enhancing synaptic plasticity and neurogenesis through activation of the PI3K/AKT/CREB/BDNF signaling pathway, and increased the expression of synaptic plasticity proteins synaptophysin (SYN) and PSD95 in hippocampal tissues (Yang et al., 2025). A combination of quercitrin and dasatinib enhances synaptic plasticity in the striatum radiatum of the cornu ammonis (CA1) region of the hippocampus in aged male rats (Krzystyniak et al., 2022). Additionally, apigenin administration alleviates cognitive disturbances by promoting hippocampal neurogenesis by increasing phosphorylation of BDNF/CREB proteins in pentylenetetrazol-treated mice (Sharma et al., 2020). Similarly, apigenin potentiates neurite outgrowth in cultured neurons by augmenting TrkB/BDNF expression in response to Aβ25-35-induced cellular stress in SH-SY5Y cells and rat cortical neurons (Gao et al., 2023). In addition, hesperidin reduced amyloid-beta accumulation and improved memory impairment by restoring hippocampal neurogenesis in 5xFAD mice via activation of AMPK/BDNF/TrkB/CREB signaling (Lee et al., 2022). Hesperidin also induced synaptic formation and hippocampal and cortical neurogenesis by enhancing the synaptogenic capacity of cortical astrocytes, mainly through increased secretion of transforming growth factor beta-1 (TGF-β1) (Matias et al., 2017). Naringin attenuated cognitive impairment through its antioxidant, modulation of cholinergic activity, and upregulation of CREB/BDNF signaling in the brains of utero-placental insufficiency (UPI) -exposed offspring (Nemati et al., 2025). Additionally, naringin reversed trimethyltin-induced cognitive dysfunction in rats by preventing neuronal loss in the CA1 region, reducing oxidative and nitrosative stress by reducing malondialdehyde (MDA) and TNFα, while elevating superoxide dismutase (SOD) and catalase activity, inhibiting expression of p-tau, Aβ, presenilin 1, and acetylcholinesterase, as well as enhancing neurogenesis and synaptogenesis (Faryadras et al., 2025).

## 5 Phytocompounds with neurogenic and plasticity-enhancing effects: clinical translation

**FIGURE 3:**
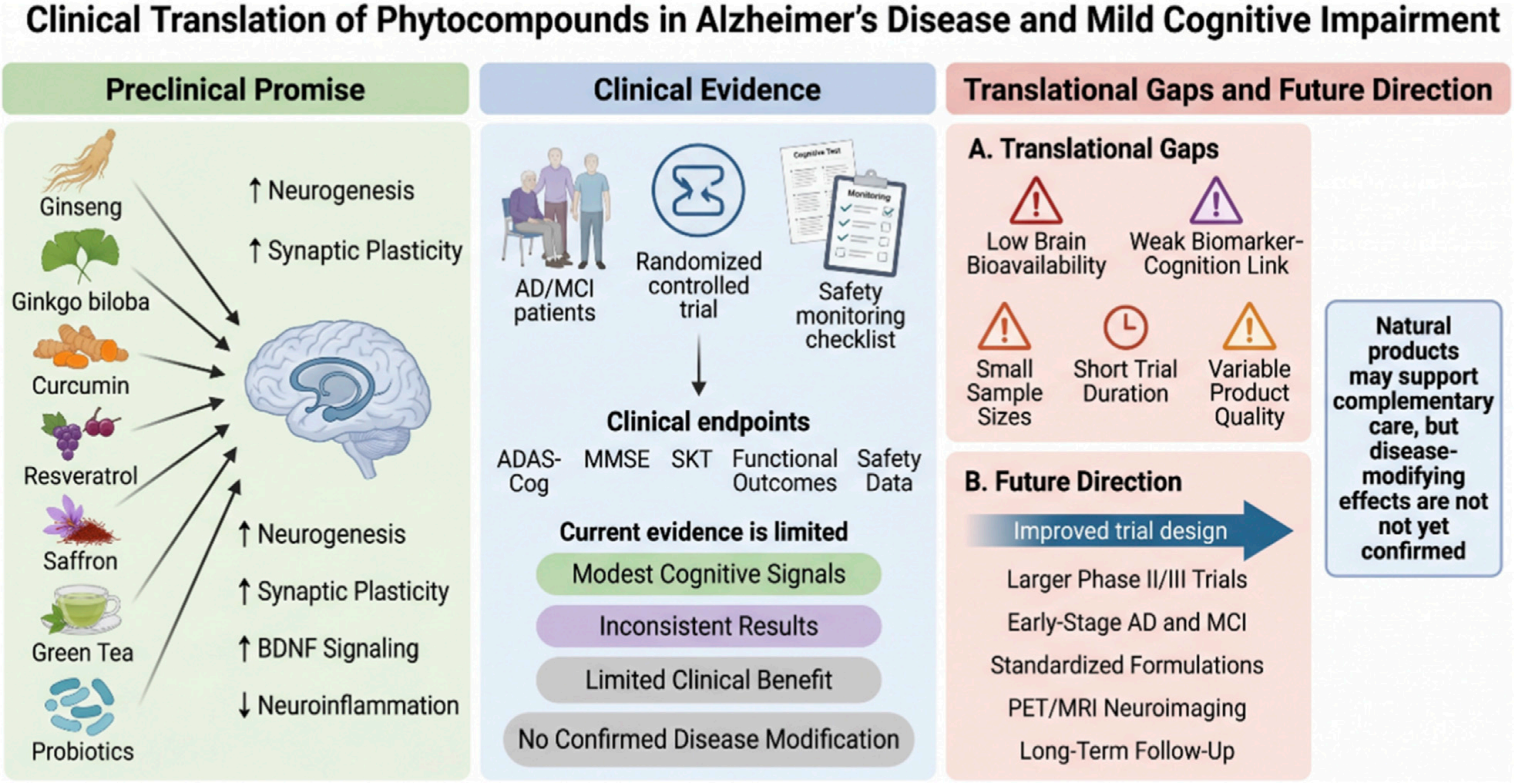
Clinical translation of neurogenic phytocompounds in AD. Clinical studies evaluating natural products with neurogenic and neuroplasticity-enhancing properties in AD and MCI show limited and inconsistent efficacy. Randomized trials investigating ginseng, *Ginkgo biloba* extract, curcumin, probiotics, saffron, and green tea derivatives report modest improvements in cognitive scores such as ADAS-Cog, MMSE, and SKT, mainly in early disease stages. However, findings are constrained by small cohorts, heterogeneous formulations, and short treatment durations. Peripheral biomarkers including BDNF may increase after supplementation but do not consistently correlate with cognitive outcomes. Although these compounds generally show good safety profiles, current evidence does not support disease-modifying effects, highlighting the need for large, long-term randomized trials with standardized formulations and robust clinical endpoints.

The Clinical Translation section offers a critical evaluation of human evidence linking the neurogenesis and neuroplasticity effects of natural products observed in preclinical studies with clinical results in AD and mild cognitive impairment (MCI) (Figure 3) (da Rosa et al., 2022; Chen et al., 2021a; Aktary et al., 2025). The evidence assessment process establishes strict requirements, permitting only data from randomized controlled trials, systematic reviews, and meta-analyses that have undergone peer review and meet high-quality standards for research on human subjects with AD or MCI. Such studies must prioritize validated cognitive endpoints such as Alzheimer’s disease assessment scale–cognitive subscale (ADAS-Cog), Mini-Mental State Examination (MMSE), and short cognitive performance test (SKT), alongside functional measurements and safety data, while regarding BDNF biomarkers as useful yet non-critical elements. Eligible trials use blinded designs, conduct intent-to-treat analyses when suitable, and feature sample sizes exceeding 30 to 50 participants with interventions lasting 12 weeks or more. Although preclinical results help define mechanistic gaps, they cannot establish efficacy. Consequently, the current findings remain highly restricted, as this research fails to demonstrate consistent disease-modifying effects and the clinical evidence supporting treatment claims is minimal, inconsistent, and mostly non-reproducible (da Rosa et al., 2022; Chen et al., 2021a; Aktary et al., 2025).

The process of developing AD therapies through natural products requires scientists to validate human evidence, since biological plausibility has consistently failed to yield effective clinical solutions. In AD, the combination of tau pathology, Aβ aggregation, and chronic inflammation fosters an environment that impairs adult hippocampal neurogenesis and synaptic plasticity; however, natural products such as probiotics, curcumin, ginsenosides, and *Ginkgo biloba* extract (GBE) demonstrate rodent model effects that fail to translate to patient outcomes. Close examination of randomized controlled trials and meta-analyses reveals modest cognitive signals of minimal strength, primarily in early-stage disease, yet these results suffer from methodological flaws, diverse demographics, and weak biomarker clinical relationships. Similarly, the use of surrogate markers such as peripheral BDNF reveals a discrepancy between mechanistic surrogates and clinical results, as these markers fail to predict cognitive or functional benefits. Overall, these findings highlight two categories, persistent scientific obstacles and research design deficiencies, that prevent successful translation while simultaneously establishing the evidence standards necessary for future clinical research (da Rosa et al., 2022; Chen et al., 2021a; Aktary et al., 2025; Hashemi et al., 2025; Kim and Cho, 2024; Mohd Sairazi and Sirajudeen, 2020).

### 5.1 Current state of clinical evidence

The study of natural products for AD and MCI has been investigated through approximately 40 randomized controlled trials since 2015. These studies focus mainly on ginseng and Ginkgo biloba, although most research uses cognition as a secondary endpoint rather than measurement of mechanism of action. The evidence is weakened because of small sample sizes typically under 100 participants, coupled with different dosing schedules, inconsistent product quality standards, and substantial differences in study outcomes. Notable systematic reviews identified approximately 31 trials enrolling 3,582 participants aged 50–90 years in interventions lasting from 8 weeks to 2 years. Considerable research has centered on ginkgo biloba extract (EGb761), with eight RCTs (n > 2,000) showing slight improvements in SKT or ADAS-Cog scores in mild-to-moderate AD after ≥24 weeks, mostly at higher doses (240 mg/day). However, large long-term studies spanning over 5 years could not demonstrate any preventive effects. For instance, saffron at 30 mg daily for 12 months slowed cognitive decline to levels comparable to memantine in moderate AD, whereas matcha green tea treatment for 12 months improved cognitive abilities and sleep quality in MCI patients; nevertheless, these results remain isolated. The two curcumin trials (NCT00099710 and NCT01001637) demonstrated positive bioavailability but yielded no cognitive benefits. Corresponding meta-analyses confirm no significant effects on MMSE or ADAS-Cog scores. Probiotics and ginseng extracts show preliminary benefits in specific subgroups with MCI. However, these findings are largely restricted to Asian cohorts and are limited by small sample sizes, brief observation periods, and heterogeneous diagnostic methods. Notably, in the few trials where minor cognitive improvements were observed, the clinical relevance remained negligible when rigorously evaluated., or, when risks are minimized, benefits are so modest as to be clinically negligible when rigorously assessed (Table 2) (da Rosa et al., 2022; Chen et al., 2021a; Aktary et al., 2025; Nahar et al., 2025; Balakrishnan et al., 2025; Inc, 2025; Ionis Pharmaceuticals, 2025; Madrid, 2025).

**TABLE 2:** Key clinical trials of natural products in AD/MCI (2015–2025).

Natural product	Trial ID/Design [reference]	n (Age range)	Dose/Duration	Primary endpoint	Key outcome	Limitations
*Ginkgo biloba* extract (EGb761)	8 RCTs/meta-analysis (Chen et al., 2021a; Aktary et al., 2025)	>2,000 (50–90)	240 mg/day, ≥24 weeks	SKT/ADAS-Cog	∼2-point SKT drop (mild AD)	Below MID (3–5 pts); no prevention in 5-year studies
Curcumin	NCT00099710 and NCT01001637 (Phase II) (Iordache et al., 2022; Lim et al., 1995)	30–100	Bio enhanced, 12–24 weeks	ADAS-Cog/MMSE	Null cognitive effect	Poor bioavailability (<1%)
Saffron	RCT (moderate AD) (Nahar et al., 2025)	∼100	30 mg/day, 12 months	Cognitive decline	Matches memantine	Isolated finding; small n
Matcha green tea	RCT (MCI) (Madrid)	<100	Standardized, 12 months	Cognition/sleep	Modest gains	Short-term; unconfirmed
Probiotics/Ginseng	Multiple RCTs (MCI) (da Rosa et al., 2022; Inc, 2025)	<100/arm	Variable, 8–52 weeks	MMSE/cognition	Early MCI hints (Asian cohorts)	Small n, ethnic bias, heterogeneity

*Summary of pivotal RCTs, highlighting inconsistent cognitive signals, small samples, and design flaws precluding disease-modification claims.

### 5.2 Cognitive and biomarker outcomes

Meta-analyses reveal that natural-product treatments for AD and MCI yield cognitive improvements with mere statistical significance, yet these fail to produce worthwhile clinical outcomes. Notably, pooled data from 37 randomized controlled trials enrolling 4,974 participants demonstrated that extended supplementation with herbal supplements, particularly Bushen capsules, led to ADAS-Cog score improvements with a standardized mean difference of −2.88. Similarly, GBE produced a two-point decrease in SKT scores in early AD, which remained below the established minimal important differences of 3–5 points and was accompanied by no consistent improvements in executive function or activities of daily living (Chen et al., 2021a; Aktary et al., 2025). Likewise, curcumin, resveratrol, and other natural compounds failed to produce any measurable effects on memory or learning, blurring the line between statistical significance and actual cognitive improvement. Moreover, Measurement differences between CSF and plasma, compounded by the restrictive nature of the blood–brain barrier, render peripheral neuroplasticity biomarkers, particularly BDNF, highly unpredictable. Although higher baseline BDNF levels clinically predict a slower MMSE decline, and interventions like probiotics and curcumin demonstrate the capacity to boost peripheral BDNF levels, these changes lack any verified, direct connection to central neurogenesis or clinically meaningful therapeutic outcomes in human patients. Consequently, these surrogate molecular signals cannot be treated as definitive proof of efficacy, and prospective human trials remain mandatory to validate whether such peripheral neurotrophic increases genuinely translate to delayed AD’s progression before yielding meaningful therapeutic results (Table 3) (Chen et al., 2021a; Aktary et al., 2025).

**TABLE 3:** Cognitive/biomarker outcomes summary.

Endpoint type	Natural product examples	Statistical effect	Clinical relevance	Biomarker correlation
Cognitive (ADAS-Cog/SKT)	GBE, Bushen capsules (37 RCTs, n = 4,974) (Chen et al., 2021a; Aktary et al., 2025)	SMD = −2.88; SKT ↓2 pts	Minimal (below MID 3–5); no executive/ADL gains	None
Memory/Learning	Curcumin, resveratrol	Null	Statistical > practical	Absent
BDNF (peripheral)	Probiotics, curcumin	WMD = 1.69 pg/mL ↑	Higher baseline predicts slower MMSE drop	Poor (plasma ≠ CSF/central)

*Disconnect between surrogate BDNF, rises and functional outcomes underscores biomarker limitations in translation.

ADAS-Cog: Alzheimer’s Disease Assessment Scale–Cognitive Subscale, SKT: short cognitive performance test, BDNF: Brain-derived neurotrophic factor, RCTs: Randomized controlled trials, GBE: *ginkgo biloba* extract, SMD: standardized mean difference, CSF: cerebrospinal fluid, WMD: weighted mean difference.

#### 5.2.1 Positioning phytochemicals in the contemporary anti-amyloid era

The modest effect sizes and the distinct disconnect between surrogate biomarker elevations and human functional capabilities underscore a critical shift in how natural products must be evaluated. These limitations highlight why plant-derived phytochemicals cannot be positioned as independent, standalone, or competitive alternatives to validated pharmaceutical interventions. The contemporary clinical landscape for managing AD has been fundamentally revolutionized by the regulatory approval of primary disease-modifying anti-amyloid therapies, exemplified by monoclonal antibodies such as lecanemab. These approved biologics are clinically validated to target the active systemic clearance of amyloid-β plaques to modify the underlying disease trajectory at its core.

In stark contrast, unstandardized natural products do not possess reproducible, regulatory-approved data confirming independent human central neurogenic repair or macroscopic structural remodeling. Therefore, to preserve scientific and clinical accuracy, phytochemicals must be realistically positioned strictly as secondary, adjunctive therapeutic strategies rather than primary therapeutic agents. Within this integrated, multi-target paradigm, optimized botanical formulations do not attempt to rival or replace monoclonal antibodies. Instead, they are intended to function as complementary components within an expanded care model, working downstream of primary biological clearance therapies to provide secondary cognitive stabilization, mitigate localized secondary oxidative stress, and support baseline synaptic proteostasis and microenvironmental maintenance.

### 5.3 Safety and feasibility considerations

Natural products demonstrate satisfactory short-term safety and tolerability profiles in elderly patients with AD and MCI. Among these, Ginkgo biloba extract appears appropriate for frail elderly populations because it exhibits a withdrawal rate of below 10%, consisting primarily of mild gastrointestinal symptoms. Similarly, the adverse-event rates for curcumin, probiotics, saffron, and matcha remain low, while adherence to treatment exceeds 80% during 12-month study periods, confirming that standardized supplement use is practical. Notably, high doses of resveratrol (2,000 mg daily for 52 weeks) elevate matrix metalloproteinase-10 levels but do not produce clinically important vital sign alterations. However, the lack of long-term safety data creates particular concern for patients with advanced AD who may experience frailty when using sedative herbs such as Melissa officinalis. Furthermore, herbal extract bioavailability varies widely across different herbal medicines, while GBE carries known interactions with anticoagulants due to its botanical properties, which complicates long-term treatment management and necessitates rigorous patient monitoring in phase III clinical trials (Table 4) (da Rosa et al., 2022; Chen X. et al., 2021; Mohd Sairazi and Sirajudeen, 2020; Ionis Pharmaceuticals, 2025; Madrid, 2025).

**TABLE 4:** Safety profiles and feasibility metrics.

Natural product	Adverse event rate vs. Placebo	Withdrawal rate	Adherence (12–52 weeks)	Long-term concerns
GBE	Mild GI (<10%) (da Rosa et al., 2022; Chen et al., 2021a)	<10%	>80%	Anticoagulant interactions
Curcumin/Probiotics/Saffron	Low/none (Mohd Sairazi and Sirajudeen, 2020)	Low	>80%	Bioavailability variability
High-dose resveratrol (2g/day)	MMP-10 ↑ (Inc.)	Minimal	Feasible	Frailty in advanced AD
Sedative herbs (e.g., Melissa)	Sedation risk (Nahar et al., 2025; Madrid)	Variable	Unclear	Lacking >1-year data

* Favorable short-term tolerability supports feasibility, but chronic data gaps demand Phase III, monitoring.

### 5.4 Synthesis of current evidence limitations and translational barriers

While preclinical investigations offer encouraging signals, the scientific evidence positioning natural products as viable treatments for neurogenesis and neuroplasticity in AD and MCI remains fundamentally incomplete. Current findings are highly restricted owing to interconnected biological factors, methodological issues, and study design limitations. To provide a rigorous, objective framework, these systemic constraints are summarized within this dedicated section.

#### 5.4.1 Methodological weaknesses in study designs

A primary obstacle in validating natural products is the lack of methodological rigor characterizing contemporary human research. Many mechanistic claims rely on underpowered experimental designs which contain less than 100 participants and use unblinded procedures. Furthermore, these studies typically last less than 24 weeks and rely entirely on subjective cognitive scales like the SKT and ADAS-Cog. Because these clinical designs consistently lack objective parameters—such as neuroimaging evaluations, functional positron emission tomography (PET) scans, or structural hippocampal volume measurements—they remain fundamentally incapable of confirming true central neurogenic repair or long-term macroscopic remodeling (Chen et al., 2021a; Hashemi et al., 2025; Snitz et al., 2009; Francis et al., 2024; Hoang et al., 2024; Horr et al., 2015).

#### 5.4.2 Biological and pharmacokinetic translational barriers

The transition from bench to bedside is severely stalled by a profound biological mismatch in experimental modeling. Preclinical research relies heavily on traditional transgenic AD mouse models, including the APP/PS1, 5xFAD, and rTg4510 strains, which are engineered to overexpress isolated, aggressive familial genetic mutations. However, these models fail to capture the multi-faceted, late-onset, and lifestyle-dependent pathobiology that characterizes over 95% of sporadic human AD cases. This model mismatch is heavily compounded by significant evolutionary divergence in central nervous system architecture between rodents and humans. Distinct differences in the cellular kinetics of adult hippocampal neurogenesis, the specific distribution of neurotrophic receptors, and localized microglial inflammatory responses mean that a phytocompound capable of rescuing synaptic plasticity in a young, genetically homogenous rodent cohort routinely fails to replicate those outcomes in a heterogeneous, elderly human population.

#### 5.4.3 Low brain Bioavailability and metabolic clearance

Even when a phytocompound demonstrates exceptional therapeutic plausibility *in vitro*, systemic administration routinely fails to yield therapeutic results due to poor pharmacokinetic properties. Highly lipophilic compounds, exemplified by curcumin, suffer from extensive first-pass hepatic metabolism and rapid systemic clearance, resulting in a central bioavailability of less than 1% at target brain tissues. Without advanced brain-targeting delivery vehicles or engineered nano-formulations, these circulating compounds are structurally unable to traverse the highly selective blood–brain barrier interface in concentrations sufficient to exert a meaningful impact on central cognitive function.

#### 5.4.4 Biomarker uncertainty and discordance

There is a profound biological disconnect between surrogate molecular signals captured in peripheral tissues and actual central neurogenesis. While clinical interventions using probiotics or herbal supplements can significantly boost peripheral plasma BDNF levels, these changes lack any demonstrated correlation to central cerebrospinal fluid (CSF) dynamics or functional cognitive outcomes. Because peripheral blood-based BDNF measurements are highly unpredictable and heavily influenced by systemic platelet release rather than localized hippocampal neurogenic niches (GZ and SVZ), this biomarker discordance demonstrates that peripheral surrogate assessments completely lack predictive power for verifying true functional or central neuroplastic recovery.

#### 5.4.5 Poor clinical reproducibility and heterogeneity

Finally, the clinical literature is highly fragmented and suffers from substantial publication bias and study design limitations. Preclinical and early-phase clinical data are heavily saturated with isolated, small-scale trials reporting exclusively positive outcomes, frequently supported by narrow industry funding or unblinded methodologies. Negative or statistically null results are rarely published, creating an artificially inflated perception of therapeutic readiness. This bias is further worsened by immense product variability; for instance, the highly inconsistent ginsenoside and flavonoid content found across unstandardized natural plant extracts prevents researchers from conducting trustworthy, reproducible meta-analyses across diverse global demographics.

**FIGURE 4:**
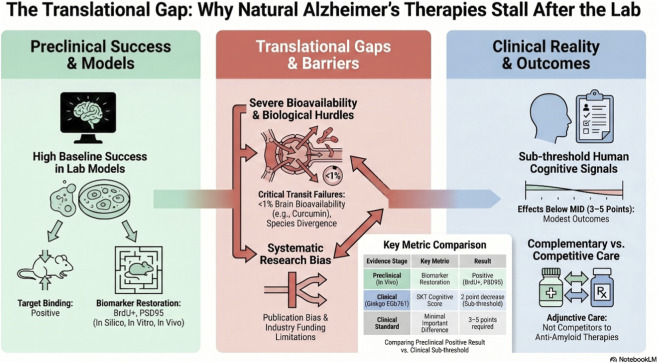
Schematic Comparison of Preclinical Evidence, Clinical Evidence, and Main Translational Gaps. The diagram contrasts the robust biochemical and behavioral markers routinely restored in controlled *in vitro* and *in vivo* animal models against the modest, sub-threshold cognitive effect sizes documented in human clinical trials. The central axis highlights the primary translational gaps including low brain bioavailability, species-specific neurogenesis differences, and biomarker uncertainty that stall the practical clinical translation of phytochemicals in Alzheimer’s disease management.

To provide a unified conceptual overview of these multi-tiered scientific barriers, a comprehensive visual synthesis is integrated below, mapping the stark contrasts within the contemporary research landscape. Figure 4 provides a detailed schematic comparison highlighting the high-success parameters characteristic of preclinical models, the restricted cognitive realities observed in human clinical evidence, and the specific translational gaps that prevent direct bench-to-bedside transition. By visually isolating the pharmacokinetic, methodological, and biological bottlenecks—such as the massive disconnect between peripheral plasma biomarkers and actual central hippocampal neurogenic niches—this schematic workflow reinforces why natural products must be framed strictly as adjunctive, stabilizing strategies rather than standalone curative interventions.

### 5.5 Implications for future clinical research

The upcoming clinical research about natural products for AD and MCI needs to conduct well-powered phase II/III randomized controlled trials which focus on either prodromal or early-stage disease patients who have MMSE scores above 20. The trials need to recruit between 200 and 300 participants for each study group in order to evaluate their effects on composite outcomes which include validated cognitive assessments and biomarkers and neuroimaging volumetric data. The ideal trial designs require standardized curcumin formulations which include Theracurmin together with pharmacokinetic optimization and patient stratification through APOE genotyping for responder identification. Mechanistic research must include central assessments of neuroplasticity via BDNF-TrkB signaling, SYN expression, and PET/MRI imaging over durations exceeding one or 2 years. Following confirmation of initial efficacy signals, head-to-head comparisons against licensed therapies such as lecanemab become essential. Until such data emerge, positioning natural products as disease-modifying treatments remains unsupported, given the absence of reproducible evidence from regulatory-approved studies; thus, they should instead function as complementary therapies (Aktary et al., 2025; Hoang et al., 2024).

High-dose GBE stands as a prime example of a natural product that may offer adjunctive cognitive stabilization in early or at-risk MCI, as evidenced by human trials. Indeed, the current standard for validating AD treatments requires both surrogate biomarkers and hard clinical outcomes to substantiate efficacy. Regrettably, there remains little evidence of enhanced neurogenesis or neuroplasticity in humans, with minimal replication across cohorts. Although the development of preventive or early-stage therapies holds potential, it requires coordinated efforts from research organizations to conduct detailed experiments bridging mechanistic signals to clinical success via integrated biomarkers and primary cognitive endpoints (Cummings et al., 2025).

## 6 Phytocompounds with neurogenic and plasticity-enhancing effects: *In silico* and network pharmacology

Curcumin, resveratrol, ginsenosides, and 7,8-DHF are examples of phytocompounds that increase BDNF and activate TrkB, which leads to downstream phosphorylation of ERK/MAPK1 and CREB to restore expression of genes linked to plasticity. Synaptic proteins are stabilized by supporting nodes such as CSNK2B, and Ca^2+^-dependent LTP is enhanced by normalization of GRIN1 (NMDA receptor subunit). These proteins work together to create a cohesive network that allows phytocompounds to prevent synapse loss associated with AD and encourage neurogenic repair.

### 6.1 Brain-derived neurotrophic factor (BDNF)

Phytochemicals boost this pathway by activating TrkB, triggering phospholipase C-gamma (PLCγ) - calmodulin-dependent protein kinase (CaMK) cascades, and enhancing synaptic translation of NMDA receptor components through heterogeneous nuclear ribonucleoprotein K regulation. Studies of the herbal formula Xiao-Chai-Hu-Tang highlight BDNF/TrkB/CREB as major molecular targets, with quercetin showing strong predicted binding and experimentally validated increases in BDNF and p-CREB expression (Chen et al., 2024). The Val66Met is a single nucleotide polymorphism in the BDNF gene known to disrupt normal intracellular trafficking and regulated secretion of the BDNF protein. Modification can decrease the amount of BDNF available at synapses, ultimately weakening mechanisms of synaptic plasticity and neuronal adaptability (Yao et al., 2020). Growing evidence suggests that the Val66Met variant may contribute to the pathophysiology of several neuropsychiatric and neurodegenerative disorders. Yao et al., has reported associations between Val66Met polymorphism and increased susceptibility to conditions such as schizophrenia and AD (Mohammadi-Khanapo et al., 2018; Park et al., 2017).

Several natural products were screened against the BDNF variant (V66M) such as Vitamin D3 (Paolacci et al., 2020). Curcumin (Jin et al., 2022) Vitamin C (Tveden-Nyborg, 2021) and Quercetin (Gao et al., 2022) ligands structures taken from PubChem database. The highest resolution *BDNF* crystallographic structure from the Protein Data Bank (PDB) database (PDB ID: 1BND) was chosen as the modeling reference (RCSB PDB, 2026) BDNF-ligand complexes were analyzed for their binding energy, interaction, stability, toxicity, and visualization using a variety of tools, including AUTODOCK VINA, BIOVIA discovery studio, PyMOL, CB-dock, IMOD server, Swiss ADEMT, and Swiss predict ligands target. Curcumin was bound to the active sites of the BDNF variant (V66M) via hydrophobic bonds, hydrogen bonds, and electrostatic interactions (Sakhawat et al., 2023).

According to Sakhawat et al. (2023), curcumin showed an average binding score of (−6.1 kj/mol). Although curcumin engages with the molecular pathway implicated in neuroplasticity; BDNF. Its therapeutic potential is significantly constrained by several pharmacokinetic barriers: (1) extremely low water solubility, yielding only ∼1% oral bioavailability; (2) extensive first-pass hepatic metabolism via glucuronidation and sulfation; and (3) limited ability to cross the blood–brain barrier without specialized delivery systems. Advanced formulations such as liposomes and nanoparticles have shown 10–100× potential increase in systemic availability and CNS uptake, although robust clinical evidence supporting these technologies is still limited (Smail et al., 2025).

### 6.2 Mitogen-activated protein kinase 1 (MAPK1/ERK2))

The MAPK pathway, especially extracellular signal-regulated kinase (ERK1/2) signaling, serves as a major convergence point for diverse phytochemical actions. When BDNF activates its receptor tyrosine kinase receptor (TrkB) it ultimately leads to the initiation of the Ras-RAF-MEK1/2-ERK1/2 cascade. Activated ERK then enters the nucleus to phosphorylate transcription factors such as CREB and Elk-1, driving expression of neuroplasticity-related genes. Importantly, pharmacological MEK inhibition completely eliminates BDNF-induced transcription in striatal neurons, underscoring the pathway’s essential role. In a study conducted by Souza et al. (2025), computational pipeline identified MAPK1 (ERK2) as a critical target (alongside CSNK2B and GRIN1) for treating neurodevelopmental disorders using phytocompounds. Epigallocatechin-3-gallate (EGCG), the major polyphenol in green tea, curcumin, resveratrol, flavonoids exerts broad neuroprotective activity by activating MAPK pathways. Computational analyses, including network pharmacology and molecular dynamics, reveal that EGCG forms highly stable complexes with its target proteins, maintaining physiologically acceptable RMSD values over long simulations. Functionally, EGCG promotes PI3K/AKT and JAK/STAT activation while suppressing TNF- and interleukin-driven neuroinflammatory pathways, underscoring its role as a polyvalent neuroprotective agent (Kamaraj, 2025). Resveratrol influences MAPK/ERK pathway, where molecular docking indicates strong binding affinity. Despite its superior blood–brain barrier penetration and measurable CNS accumulation, resveratrol’s therapeutic impact is limited by its very low oral bioavailability (∼0.5–3%), which restricts effective systemic concentrations (Hedayati et al., 2025).

### 6.3 cAMP response element-binding protein (CREB)

CREB is used as a key transcription in which phytocompounds drive neurogenesis in AD relevant models. Curcumin promotes neuroplasticity through at least five complementary mechanisms. It boosts BDNF–TrkB–CREB signaling, suppresses NF-κB/MAPK-driven inflammation, and chelates dysregulated copper and zinc, a key factor in Alzheimer-related neurotoxicity. It also inhibits amyloid-beta aggregation and enhances antioxidant defenses by activating the NRF2/ARE pathway. Together, these actions position curcumin as a broad-spectrum modulator of neuronal resilience and repair (Chen et al., 2021b). In mice, curcumin was found to increase NSC proliferation and improve cognitive functions. Accompanied by activation of pro-survival and plasticity pathways through having high affinity to upstream CREB-activating proteins-including ERK2/MAPK1 (Chen et al., 2025; Obulesu and Lakshmi, 2014; Magistri et al., 2015; Nguyen et al., 2024). It has been difficult to validate docking scores or MD simulations of natural products binding directly to CREB, due to CREB’s intrinsically disordered *N*-terminal region and lack of a well-defined small-molecule binding pocket. Network pharmacology analyses identify the BDNF/TrkB/CREB pathway as a central mechanism of Xiao-Chai-Hu-Tang. Molecular docking shows that its key flavonoids especially quercetin bind strongly to regulatory proteins within this pathway. Experimental studies further validate these predictions, demonstrating increased BDNF and phosphorylated CREB expression in hippocampal neurons following treatment (Feng et al., 2025).

### 6.4 Tropomyosin kinase receptor B binders (TrkB)

The BDNF–TrkB axis is the primary regulatory system governing neurogenesis, synaptic plasticity, and cognition. When TrkB is activated, it recruits adaptor proteins and stimulates PLCγ-dependent IP_3_ signaling, increasing intracellular calcium and triggering CaMK-mediated plasticity pathways.

Network pharmacology studies of Xiao-Chai-Hu-Tang highlight BDNF/TrkB/CREB as central therapeutic targets. Docking analyses show strong interactions between its major flavonoid quercetin and pathway regulators. Experimental work confirms these predictions, demonstrating increased BDNF and phosphorylated CREB expression in hippocampal neurons (Feng et al., 2025). *Panax ginseng*–derived ginsenosides are among the best-characterized phytochemicals with demonstrated neurogenic and neuroprotective effects across diverse animal models. Their actions center on activating the PI3K/AKT and MAPK/ERK pathways, which in turn elevate BDNF levels and enhance TrkB-dependent synaptic plasticity. Network pharmacology and reverse virtual screening identify ginsenosides as multitarget ligands, capable of stimulating NRF2-ARE antioxidant responses while simultaneously suppressing NF-κB–mediated inflammation. Despite strong preclinical evidence, clinical translation is hindered by low oral bioavailability and restricted blood–brain barrier penetration, with optimized formulations achieving only modest hippocampal accumulation relative to plasma (Huang et al., 2019). Epigallocatechin-3-gallate (EGCG), the major green tea polyphenol, exerts potent neuroprotective effects through multiple mechanisms through modulation of BDNF–TrkB signaling. Network pharmacology combined with molecular dynamics demonstrates that EGCG forms stable protein–ligand complexes, maintaining physiologically acceptable RMSD values over extended simulations. Functionally, EGCG activates PI3K/AKT and JAK/STAT pathways while suppressing TNF- and interleukin-driven neuroinflammation, underscoring its role as a polyvalent neuroprotective agent (Alam et al., 2024).

### 6.5 Casein kinase 2, beta subunit (CSNK2B)

Casein kinase II (CKII)-whose β-subunit is encoded by CSNK2B-is gaining attention as a potential modulator of neuroplasticity, CSNK2B encodes the regulatory β subunit of casein kinase 2 (CK2), a constitutively active serine/threonine kinase that modulates survival and synaptic signaling pathways linked to CREB (Borgo et al., 2021). CSNK2B role in phytochemical-based neuroprotection is still poorly defined. Network pharmacology studies incorporating CSNK2B as a target remain limited, but computational screening suggests that flavonoids such as quercetin and kaempferol may modulate CKII through allosteric interactions. Experimental confirmation of these predicted interactions, however, is still lacking. Other docking studies suggest that curcumin and resveratrol can bind CSNK2B with moderate affinity (ΔG −6.4 to −7.2 kcal/mol). However, cell-free kinase assays reveal only 15%–25% inhibition at physiological concentrations—far below what docking geometry would predict highlighting the persistent gap between *in silico* predictions and actual biochemical activity (Toader et al., 2025).

### 6.6 Glutamate ionotropic receptor NMDA type subunit 1 (GRIN1)

GRIN1 (GluN1) forms the obligatory subunit of NMDA receptors, assembling with GluN2A or GluN2B to create calcium-permeable channels essential for long-term potentiation, developmental plasticity, and learning. Recent structural analyses show that even subtle ligand-binding domain perturbations in GluN1—particularly around the glycine-binding pocket—can markedly alter agonist affinity and receptor gating, contributing to synaptic dysfunction and NMDA-related pathologies, including Alzheimer’s disease (Montanucci et al., 2025). Phytochemicals modulate NMDA-dependent plasticity through allosteric mechanisms, direct interactions with regulatory domains, and indirect enhancement via BDNF–TrkB signaling, which increases synaptic NMDA receptor expression. Because NMDA overactivation drives calcium-mediated excitotoxicity, effective phytochemical modulation must maintain a functional balance—enhancing plasticity without promoting pathological receptor overdrive, a balance that single-target interventions often fail to achieve (Korinek et al., 2024). Emerging evidence suggests that epigallocatechin-3-gallate (EGCG) interacts directly with NMDA receptors, particularly the GluN1 subunit, supporting its role as a multimodal neuroprotective agent. While primary literature provides docking scores and molecular-dynamics-validated binding for several natural ligands at NMDA receptor structures, a quantitative, experimentally confirmed link between specific Alzheimer’s-relevant phytochemicals and GRIN1 binding remains an open area for targeted *in silico* and *in vitro* investigation (McCalley et al., 2014; Hsieh et al., 2021).

## 7 Phytocompounds with neurogenic and plasticity-enhancing effects: formulations

### 7.1 The blood–brain barrier: a major obstacle to central nervous system drug transport

The blood–brain barrier (BBB) represents a highly specialized and selectively permeable interface that regulates the exchange of substances between the systemic circulation and the brain. Its primary role is to shield neural tissue from harmful molecules while permitting the controlled entry of essential nutrients required for normal brain activity (Ahmed et al., 2026a; Shabbir et al., 2020). Structurally, the BBB is composed mainly of brain endothelial cells that interact with several supporting components, including pericytes, astrocytes, brain capillaries, and the basement membrane. A defining characteristic of BBB endothelial cells is their close arrangement and the presence of specialized intercellular junctions that maintain barrier integrity. These junctions include tight junctions and adherens junctions. Tight junctions play a central role in strengthening trans-endothelial resistance, preserving cellular polarity, and regulating leukocyte trafficking, whereas adherens junctions contribute to structural stability and intercellular adhesion, thereby sustaining the essential architecture of the BBB (Ahlawat et al., 2020; Alba et al., 2025). Due to this intricate structural organization, the BBB serves as a formidable boundary that limits the penetration of circulating compounds into the brain’s extracellular environment. Consequently, nearly 98% of therapeutic agents and many diagnostic molecules are unable to traverse this barrier. This strict selectivity represents a major challenge in the development of effective pharmacological strategies for neurological disorders, including AD (Manek et al., 2020; Farag et al., 2025).

### 7.2 Advanced brain-targeting strategies in AD

#### 7.2.1 Intranasal delivery

Intranasal drug administration is an attractive strategy for brain targeting because it can bypass the blood–brain barrier (BBB) and provide a direct pathway to the central nervous system. Drugs delivered through the nasal cavity may reach the brain via the olfactory and trigeminal neural pathways, enabling faster and more efficient transport compared with conventional systemic delivery (Ibrahim et al., 2024; Elsharkawy et al., 2023). Traditional nasal formulations such as sprays, drops, gels, and powders can support both local and systemic treatment. To enhance drug absorption, permeation enhancers and mucoadhesive polymers are often incorporated to improve drug permeability and prolong residence time within the nasal cavity (Ahmed et al., 2026b; Fahmy et al., 2026). In addition, nanocarrier-based systems have been widely investigated to protect drug molecules, improve mucosal penetration, and enable sustained drug delivery to the brain. Overall, nose-to-brain delivery offers a non-invasive and efficient platform for improving the therapeutic effectiveness of drugs used in AD while reducing systemic exposure (Zhang et al., 2025b; Taha et al., 2023).

#### 7.2.2 Transdermal delivery

Transdermal drug delivery systems provide a non-invasive method for systemic drug administration through the skin. These systems allow controlled and sustained drug release while avoiding gastrointestinal degradation, enzymatic metabolism, and first-pass hepatic metabolism (Jeong et al., 2021). Drug transport across the skin can occur through passive diffusion or by using active enhancement techniques such as electrical, mechanical, or thermal methods to increase permeability (Tariot et al., 2022). For AD therapy, transdermal delivery offers several advantages, including improved dosing consistency, reduced systemic side effects, and enhanced patient compliance compared with conventional oral administration. By maintaining stable drug levels in the bloodstream, transdermal systems may provide a more reliable and convenient approach for long-term management of neurodegenerative disorders (Tawfik et al., 2023).

#### 7.2.3 Ultrasound-mediated delivery

Ultrasound-assisted drug delivery has emerged as a promising non-invasive technique for enhancing drug penetration and targeted delivery to brain tissues. Ultrasound waves can induce mechanical and thermal effects that temporarily increase tissue permeability and facilitate drug transport across biological barriers such as the BBB (Eklund et al., 2021). When combined with responsive nanocarriers, ultrasound can trigger localized drug release at the target site, improving therapeutic precision while minimizing systemic exposure. In addition, ultrasound-based systems can be integrated with imaging technologies, enabling simultaneous drug delivery and disease monitoring. Overall, this approach provides a minimally invasive and controllable strategy for improving brain drug delivery in AD treatment (Fonseca et al., 2021).

### 7.3 Nanotechnology in AD diagnosis

Nanotechnology has introduced new tools for the early detection and monitoring of AD. Various nanomaterials have been developed to identify disease-related biomarkers and improve understanding of the molecular mechanisms involved in AD progression. Compared with traditional diagnostic techniques, nano-based biosensors provide higher sensitivity, improved specificity, and the possibility of real-time monitoring. However, their clinical use is still limited because nanoparticles often have complex pharmacokinetic behavior and short circulation time in the bloodstream (Panghal and Flora, 2024).

#### 7.3.1 Magnetic nanoparticles for Alzheimer’s imaging

Magnetic nanoparticles have been widely explored for imaging Aβ plaques, which are a major pathological feature of AD. Studies have shown that iron accumulates within amyloid plaques as magnetic nanocrystals with super-paramagnetic properties that can assist in disease detection (Plascencia-Villa et al., 2016). Based on this concept, different magnetic nanoprobes have been designed as contrast agents capable of crossing the blood–brain barrier and selectively binding to amyloid deposits (Cheng et al., 2015). The targeting ability of these nanoprobes can be improved by attaching antibodies or peptides that recognize Aβ or tau proteins, enabling more specific detection of AD biomarkers (Li et al., 2016). Surface modifications may also enhance their binding affinity and imaging accuracy (Nasr et al., 2018). These bio-functionalized magnetic nanoparticles have shown promising results in magnetic resonance imaging (MRI) for identifying amyloid plaques *in-vivo* (Wadghiri et al., 2013). In addition to plaque imaging, magnetic nanoprobes have been explored for detecting other potential biomarkers associated with AD pathology. Some nanostructures combine magnetic and fluorescent properties, allowing multimodal imaging that improves visualization of pathological changes (Skaat and Margel, 2009). Advanced nanoprobe systems integrating magnetic and fluorescence signals can also provide sensitive detection of Aβ species in experimental models (Zeng et al., 2018). Furthermore, nanoparticles functionalized with antibodies targeting Aβ oligomers (AβO) have demonstrated strong selectivity for early-stage disease detection and may help evaluate therapeutic responses during drug development (Viola et al., 2015).

#### 7.3.2 Quantum dot-based biosensors

Quantum dots (QDs) are fluorescent semiconductor nanoparticles with unique optical properties, including broad excitation ranges, narrow emission spectra, and strong fluorescence intensity (Wagner et al., 2019). These characteristics make them valuable tools for imaging and biosensing applications (Gil et al., 2021). In addition to fluorescence detection, QDs can also be measured electrochemically, which expands their diagnostic capabilities (Zhang et al., 2022b). QDs can be functionalized with biological molecules such as antibodies, enzymes, or DNA, allowing them to detect AD-related biomarkers with high sensitivity (Mars et al., 2018). As a result, QD-based biosensors have been developed to identify biomarkers such as Aβ peptides and ApoE variants in biological samples (Zhao et al., 2022). Importantly, QD systems can also be designed for simultaneous detection of multiple biomarkers, which is important for improving diagnostic accuracy in complex diseases like AD. In several studies, QD-based detection methods showed better sensitivity than conventional fluorescent dyes (Morales-Narvaez et al., 2012). Despite these advantages, some limitations remain. Many QDs contain toxic heavy metals, may exhibit unstable fluorescence signals, and are relatively large compared with organic fluorophores (Zhong et al., 2021). In addition, their high cost and technical challenges related to detection equipment limit their widespread clinical application (Torres et al., 2023).

#### 7.3.3 Real-time nanosensors for Alzheimer’s diagnosis

**FIGURE 5:**
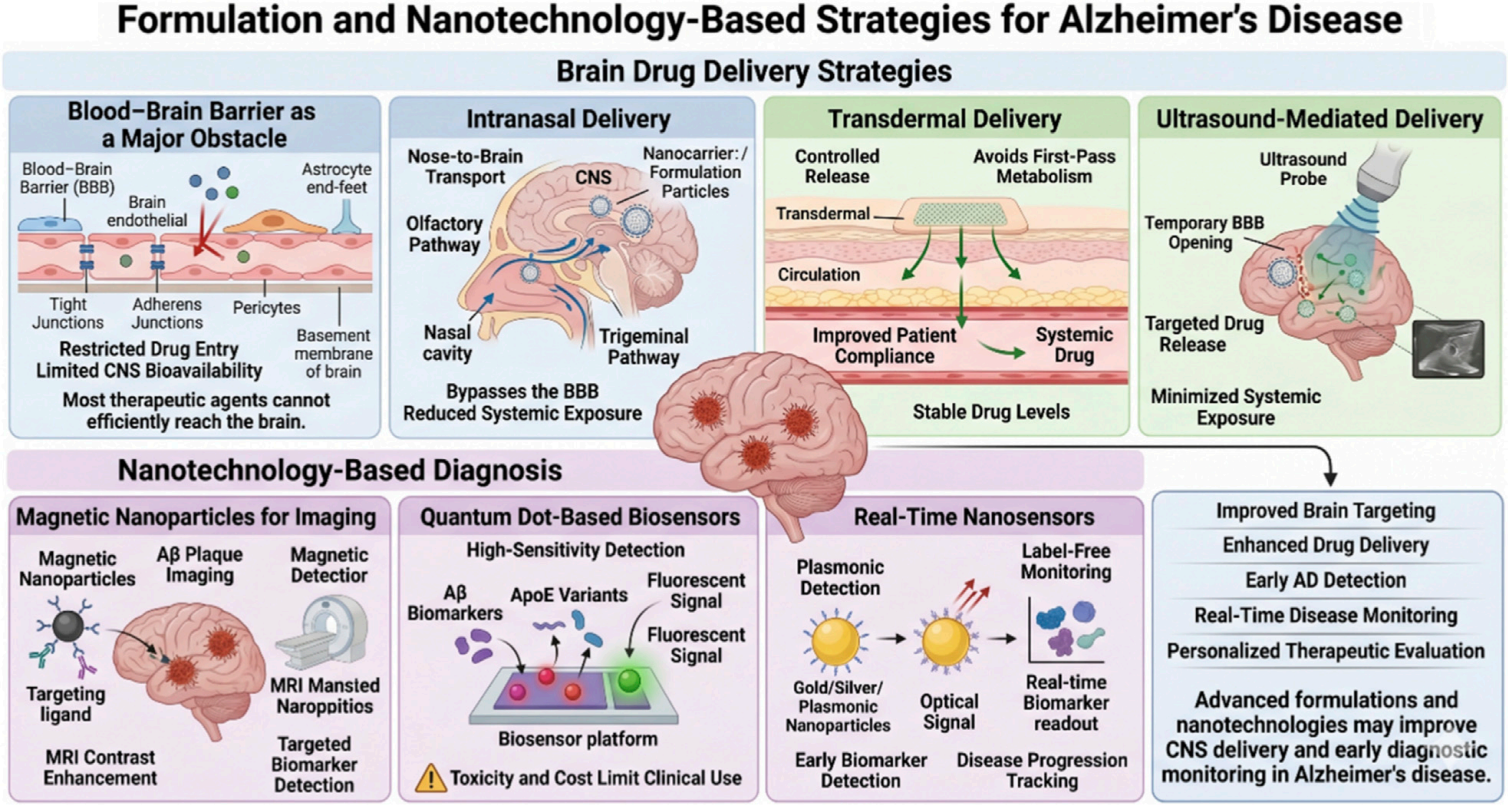
Advanced delivery and nanotechnology strategies for brain targeting in AD. Limited blood–brain barrier (BBB) permeability restricts delivery of many neurogenic phytocompounds in AD. Advanced strategies aim to improve brain targeting and diagnostic detection. Intranasal delivery enables direct nose-to-brain transport via olfactory and trigeminal pathways, while transdermal systems provide sustained systemic delivery and improved compliance. Ultrasound-mediated approaches transiently increase BBB permeability to enhance drug penetration. In addition, nanotechnology-based platforms support AD diagnosis and monitoring. Magnetic nanoparticles allow MRI detection of Aβ plaques, whereas quantum dot biosensors and plasmonic nanosensors enable highly sensitive detection of AD biomarkers such as Aβ and ApoE. Together, these technologies enhance brain-targeted delivery and biomarker detection for phytochemical-based AD interventions.

Recent progress in nanosensor technology has enabled real-time detection of AD biomarkers, which may improve early diagnosis and disease monitoring. Nanomaterials are particularly useful for biosensing because of their high surface area, ability to bind biological molecules, and strong signal amplification properties (Carneiro et al., 2020). Plasmonic nanoparticles such as gold, silver, and platinum exhibit a phenomenon known as localized surface plasmon resonance, which allows sensitive detection of molecular interactions by monitoring changes in optical signals (Palaniyandi et al., 2023). This technique can detect protein aggregation processes associated with AD and track biomarker interactions in a label-free and real-time manner (Elbassal et al., 2017). Nanotechnology-based biosensors have also been developed to detect important AD biomarkers such as Aβ peptides and ApoE-related interactions at extremely low concentrations in biological fluids. These advanced sensing platforms may support early diagnosis, improve understanding of disease progression, and help evaluate the effectiveness of therapeutic interventions (Nair et al., 2020). Figure 5 summarizes different nanotechnology-based diagnostics.

### 7.4 Nano-formulations and phytochemical-based approaches for neurodegenerative disorders

Nanotechnology provides versatile platforms for drug delivery and neuro-therapeutics, improving drug stability, brain penetration, controlled release, and functional monitoring (Pramoda et al., 2025). Several classes of nanomaterials have been explored for neurological applications, each with unique advantages.

#### 7.4.1 Lipid-based nanocarriers

Lipid-based drug delivery systems, including liposomes, nanoemulsions, solid lipid nanoparticles, nanostructured lipid carriers and cubosomes, provide versatile platforms for encapsulating both hydrophilic and hydrophobic drugs. These carriers allow sustained release, high bioavailability, and BBB penetration. Among them, cubosomes offer biocompatibility and bioadhesive properties, and they can be engineered to overcome limitations such as poor drug loading (Ahmed et al., 2025; Ragheb et al., 2026).

#### 7.4.2 Carbon-based nanostructures

Carbon nanotubes (CNTs) are cylindrical nanostructures composed of carbon atoms, notable for their high surface area, electrical conductivity, and mechanical strength (Qian et al., 2012). These properties allow CNTs to function as scaffolds in neuroengineering, supporting neuronal differentiation, axonal growth, neuroprotection, and electrical stimulation. CNTs can respond to external stimuli such as pH, temperature, magnetic fields, and electric signals, making them suitable for controlled drug release and neural interfacing. Electrospun nanofibers are emerging as safer alternatives to CNTs for neuroprosthetics and brain tissue engineering. Their porous structure mimics the extracellular matrix, enhancing cell adhesion and differentiation while minimizing immunogenicity and environmental risks. CNTs and nanofibers can be used alone or combined with biodegradable polymers to form hybrid scaffolds for deep brain stimulation, neural repair, or targeted drug delivery (Chowdhury et al., 2025).

#### 7.4.3 Polymeric nanoparticles, nanocapsules, and nanospheres

Polymeric nanoparticles are solid colloidal systems made from natural or synthetic polymers (e.g., PLGA), capable of encapsulating drugs to protect against degradation and improving penetration into the CNS. These nanoparticles avoid rapid clearance by macrophages and extend systemic circulation, increasing the likelihood of crossing the BBB. Nanocapsules consist of a drug-loaded core surrounded by a polymeric shell, providing controlled and sustained drug release. In contrast, nanospheres are matrix systems in which the drug is uniformly dispersed throughout the polymer network. Both systems can be engineered for size, surface charge, and functionalization with targeting ligands (e.g., peptides or antibodies), enhancing selective delivery to neuronal tissues (Teama et al., 2025; Cirmi et al., 2016).

#### 7.4.4 Nanogels and nanosuspensions

Nanogels are hydrophilic, crosslinked polymeric networks (20–200 nm) capable of high drug loading (40%–60%). Their soft, water-rich structure allows improved diffusion of oligonucleotides or peptides into brain tissues while reducing liver and spleen uptake. Nanogels can also be functionalized to respond to stimuli like pH or temperature, enabling site-specific release. Nanosuspensions contain crystalline drug particles stabilized with surfactants or lipid coatings, offering enhanced solubility, bioavailability, and uniform drug distribution. These systems are easy to administer and can improve therapeutic efficacy for poorly soluble neuroactive compounds (Singh et al., 2016; Chavhan, 2025).

#### 7.4.5 Polymeric nanoliposomes

Nanoliposomes are vesicular systems composed of phospholipid bilayers, forming hydrophilic cores and hydrophobic bilayers that can accommodate a wide range of drugs. Surface modifications (e.g., PEGylation) reduce opsonization and hepatic clearance, prolonging circulation time. Nanoliposomes have demonstrated efficient BBB penetration and are suitable for targeted CNS drug delivery, making them ideal for neurodegenerative therapies (Talens-Visconti et al., 2022).

#### 7.4.6 Exosomes

Exosomes are natural extracellular vesicles (30–150 nm) produced by eukaryotic cells. Their biocompatibility, small size, ability to cross biological barriers, and targeted delivery capacity make them promising carriers for drugs in neurodegenerative diseases. Exosomes can either propagate pathogenic proteins or deliver therapeutic molecules across the BBB, making them valuable both as treatment vehicles and diagnostic tools (Rehman et al., 2019; Strathearn et al., 2014).

#### 7.4.7 Green nanoparticles

Biogenic nanoparticles synthesized using plant extracts (e.g., gold, silver, selenium, copper) offer neuroprotective effects. For instance, silver nanoparticles combined with antioxidant-rich pomegranate extract showed protection against neurotoxicity in human neuronal cells, highlighting the therapeutic potential of phytochemical-based green nanomaterials in neurodegenerative disorders (Chowdhury et al., 2025).

## 8 Conclusion

In conclusion, impaired neurogenesis and neuroplasticity play an important role in the pathophysiology of Alzheimer`s disease (AD), contributing considerably to cognitive decline and synaptic dysfunction. Preclinical studies showed that natural products can be promising multi-target therapeutic candidates that can improve neuronal regeneration, synaptic connectivity, and cognitive function by modulating key molecular pathways such as BDNF/TrkB, CREB, and PI3K/Akt signaling. Phytochemicals such as ginsenosides, EGCG, curcumin, resveratrol, and flavonoids exhibit notable neurogenic and neuroplasticity enhancing effects by various mechanisms including antioxidant, anti-inflammatory and neurotrophic pathways. Despite the promising outcomes of pre-clinical studies on natural products, their clinical studies remain limited due to poor bioavailability, inconsistent efficacy, and methodological constraints in human studies. Future research should focus on well-designed clinical trials evaluating natural products, advanced delivery systems, and biomarker-guided techniques to evaluate their clinical potential in AD management.

## Glossary

5-HT1A: 5-hydroxytryptamine receptor
5-HT2: 5-hydroxytryptamine (serotonin) receptors
AD: Alzheimer’s disease
**ADAS-Co**g: Alzheimer’s Disease Assessment Scale–Cognitive Subscale
Akt: Protein kinase B
AMPK: AMP-activated protein kinase
APOE: Apolipoprotein E
APP: Amyloid precursor protein
Aβ: Amyloid-β
AβO: Aβ oligomers
BBB: Blood-brain barrier
Bcl-2: B-cell lymphoma two
BDNF: Brain-Derived Neurotrophic Factor
CA1: Cornu ammonis
CaMK: calmodulin-dependent protein kinase
CIN: Chromosome Instability
CKII: Casein kinase II
CNTs: Carbon nanotubes
CREB: Cyclic AMP response element-binding protein
CSF: Cerebrospinal Fluid
CSNK2B: Casein Kinase 2, Beta Subunit
DG: Dentate gyrus
EGCG: Epigallocatechin gallate
EGR-1: Early growth response protein 1
ERK: Extracellular regulated kinase
GBE: *Ginkgo biloba* extract
GRIN1: Glutamate Ionotropic Receptor NMDA Type Subunit 1
GSK3β: Glycogen synthase kinase-3β
HFFD: High-fat and high-fructose diet
IRS-1: Insulin receptor substrate-1
JNK: c-Jun-N-terminal kinase
LTD: long-term depression
LTP: long-term potentiation
MAPK1: Mitogen-activated protein kinase 1
MCI: Mild cognitive impairment
MD: molecular dynamics
MDA: Malondialdehyde
MMSE: Mini-Mental State Examination
mTOR: Mammalian target of rapamycin
NEP: Neprilysin
NF-κB: Nuclear factor kappa B
NGF: Nerve growth factor
NMDA: N-methyl-D-aspartate
NSCs: Neural stem cells
NT-3: Neurotrophin 3
PDB: Protein Data Bank
PET: positron emission tomography
PI3K: Phosphoinositide 3-kinase
PKC: Protein kinase C
PLCγ2: Phospholipase Cγ2
PS1: Presenilin-1
PSD95: Postsynaptic density protein 95
QDs: Quantum dots
RCTs: Randomized Controlled Trials
rTg4510: Regulatable Transgenic 4,510
SGZ: sub granular zone of the dentate gyrus
SIRT1: Sirtuin-1
SKT: Short Cognitive Performance Test
SNARE: soluble N-ethylmaleimide-sensitive factor attachment protein receptor
SOD: Superoxide dismutase
SVZ: sub ventricular lining the lateral ventricles zone
SYN: Synaptophysin
TGF-β1: Transforming growth factor beta-1
TNF-α: Tumor necrosis factor-alpha
TrkB: Tropomyosin receptor kinase B
UPI: utero-placental insufficiency
Val66Met: Valine-66-Methionine
wnt: Wingless-related kinase
β-catenin: Beta-catenin
